# Chromosome-level genome assembly of the freshwater mussel *Sinosolenaia oleivora* (Heude, 1877)

**DOI:** 10.1038/s41597-024-03451-5

**Published:** 2024-06-08

**Authors:** Xueyan Ma, Wu Jin, Wanwen Chen, Qian Liu, Haizhou Jiang, Yanfeng Zhou, Pao Xu, Haibo Wen, Dongpo Xu

**Affiliations:** 1 Key Laboratory of Integrated Rice-Fish Farming Ecology, Ministry of Agriculture and Rural Affairs, Freshwater Fisheries Research Center, Chinese Academy of Fishery Sciences, Wuxi, 214081 China; 2 Sino-US Cooperative Laboratory for Germplasm Conservation and Utilization of Freshwater Mollusks, Freshwater Fisheries Research Center, Chinese Academy of Fishery Sciences, Wuxi, 214081 China; 3 Wuxi Fisheries College, Nanjing Agricultural University, Wuxi, 214081 China

**Keywords:** Eukaryote, Ichthyology

## Abstract

*Sinosolenaia oleivora* (Bivalve, Unionida, Unionidae), is a near-endangered edible mussel. In 2022, it was selected by the Ministry of Agriculture and Rural Affairs as a top-ten aquatic germplasm resource, with potential for industrial development. Using Illumina, PacBio, and Hi-C technology, a high-quality chromosome-level genome of *S. oleivora* was assembled. The assembled *S. oleivora* genome spanned 2052.29 Mb with a contig N50 size of 20.36 Mb and a scaffold N50 size of 103.57 Mb. The 302 contigs, accounting for 98.41% of the total assembled genome, were anchored into 19 chromosomes using Hi-C scaffolding. A total of 1171.78 Mb repeat sequences were annotated and 22,971 protein-coding genes were predicted. Compared with the nearest ancestor, a total of 603 expanded and 1767 contracted gene families were found. This study provides important genomic resources for conservation, evolutionary research, and genetic improvements of many economic traits like growth performance.

## Background & Summary

Freshwater mussels (Unionoida) represent the most diverse order of freshwater bivalves^1^ and are found in all regions of the world except the Antarctic^2^. They not only play an important role in the food web structure and material cycle of ecosystems^3,4^ but also have high economic value, such as for food^5^, pearl cultivation^6^, and anti-tumor ingredients^7^. They also have been used as an indicator for biological monitoring and evaluation of heavy metal pollution^8^.

Freshwater mussels are benthic filter feeders^9^. Suitable substrate, water quality, and food are important factors for the survival and reproduction of mussels. In recent years, human activities, such as river diversion, chemical pollution, and overfishing have caused serious damage to mussel habitats^10^. The developmental life history of most mussels involves a parasitic larval stage (glochidia) that must attach to vertebrate hosts (primarily fish) to complete metamorphosis^11^ which increases their vulnerability^2^. The International Union for Conservation of Nature (IUCN) Red List reports that 173 species are extinct, endangered, or threatened, 99 are vulnerable or nearly threatened, and 84 are unclassified because data are deficient^12^.

There are 57 endemic species in China^13^, and eight species have now been listed as Grade II national protected animals^14^. The biodiversity and population size of freshwater mussels in large water bodies such as the Yangtze River^15^ and the Songhua River^16^ have shown a significant decline. S.*oleivora* is endemic to China. In 2022, *S. oleivora* was identified as one of the top ten characteristic aquatic germplasm resources by the Ministry of Agriculture and Rural Affairs. *S. oleivora* has fresh and tender meat, delicious taste, and high nutrient content^17^. In Fuyang of Anhui Province, Tianmen of Hubei Province, and other places, *S. oleivora* is a famous delicacy with a high economic value, and it is called “abalone in Huaihe River.” It once ranged an extensive distribution—in five freshwater lakes and the tributaries of the Yangtze and Huaihe Rivers^18^. Habitat fragmentation and other human activities (e.g., overfishing) have resulted in their endangerment^19^. Tianmen in Hubei Province and Fuyang in Anhui Province has established the *S. oleivora* Nature Reserve to support this ecologically and economically vital resource.

Genomic data is considered fundamental for revealing biological characteristics, inferring evolutionary mechanisms, and promoting effective conservation^20^. To date, only seven freshwater mussel species have had their genomes sequenced (Table S1, Supplementary File)^21–28^, and only one of these is a Chinese species^27^. The whole genome of *S. oleivora* is lacking. We applied multiple sequencing technologies, including Illumina Nova 6000 sequencing, PacBio long-read sequencing (PacBio), and high-throughput chromosome conformation capture (Hi-C) technology to complete genome sequencing and assembly. Three methods, including *de novo* gene prediction, homolog, and RNA-Seq-based prediction, were used to perform genomic annotation. In addition, the comparative genomics analysis of *S. oleivora* and 10 other distantly related species was performed. This study provides important genomic resources for conservation and evolutionary research and guides genetic trait improvements (e.g., growth).

## Methods

### Sample collection and sequencing

**Fig. 1:**
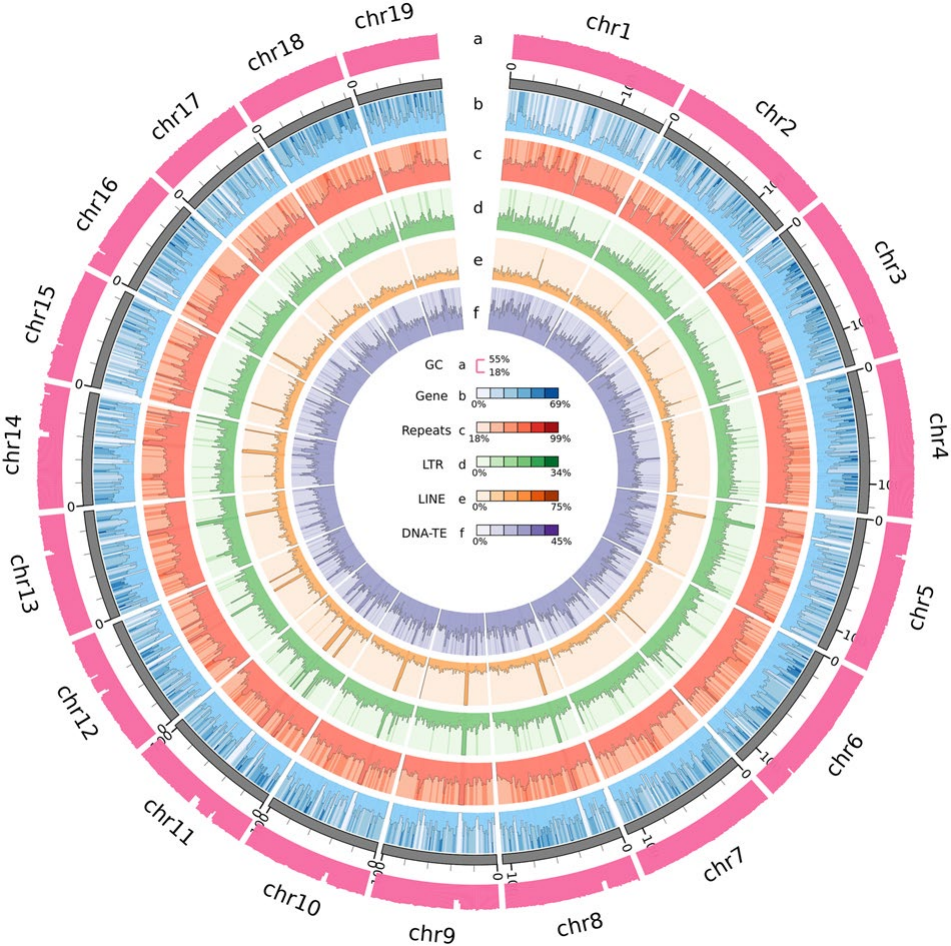
Genome characteristics of *Sinosolenaia oleivora*.

One female *S. oleivora* was sampled from the national-level protection zone of the aquatic germplasm resource of *S. oleivora* in the Fuyang Division of Huaihe River (32.428725°N, 115.600287°E). Total DNA was extracted from the adductor muscle of *S. oleivora* using the DNeasy Blood and Tissue Kit (Qiagen, Germany) for genome sequencing. For short-read sequencing, Covaris M220 was used to break DNA into 300–350 bp fragments. DNA library preparation was completed by terminal repair, an A-tail addition, sequencing junction addition, DNA purification, and bridge PCR. Based on a paired-end(PE) sequencing strategy. These libraries were sequenced on the Illumina NovaSeq Nova 6000 platform. For long-read sequencing, according to the PacBio standard protocol, a PacBio HiFi library was generated using an SMRTbell Template Prep Kit 2.0 (Pacific Biosciences, USA) and sequenced using the PacBio Sequel II platform. A Hi-C library was prepared following the Hi-C library protocol^29^ and sequenced using the Illumina Novaseq 6000 platform. Total RNA was extracted from the adductor muscle of *S. oleivora* using TRIzol reagent (Invitrogen, MA, USA) for transcriptome sequencing. The RNA-seq library was generated using NEBNext^®^Ultra^TM^ RNA Library Prep Kit (NEB, USA) for PE sequencing, and short reads were produced on the Illumina NovaSeq 6000 platform. A total of 192.1 Gb of Illumina data, 63.2 Gb of PacBio data, 191.8 Gb of Hi-C data, and 5.6 Gb RNA-Seq data were obtained (Fig. 1, Table 1).

**Table 1:** Statistics for the sequencing data of the *Sinosolenaia oleivora* genome.

Type	Library size (bp)	Raw data (Gb)	Clean data (Gb)	Coverage (×)
Illumina Nova	350	217.6	192.1	106.15
PacBio SMRT	15k	107.3	63.2	30.83
Hi-C	350	197.5	191.8	96.34
Illumina RNA-Seq	350	5.9	5.6	

### Estimation of genome size

**Fig. 2:**
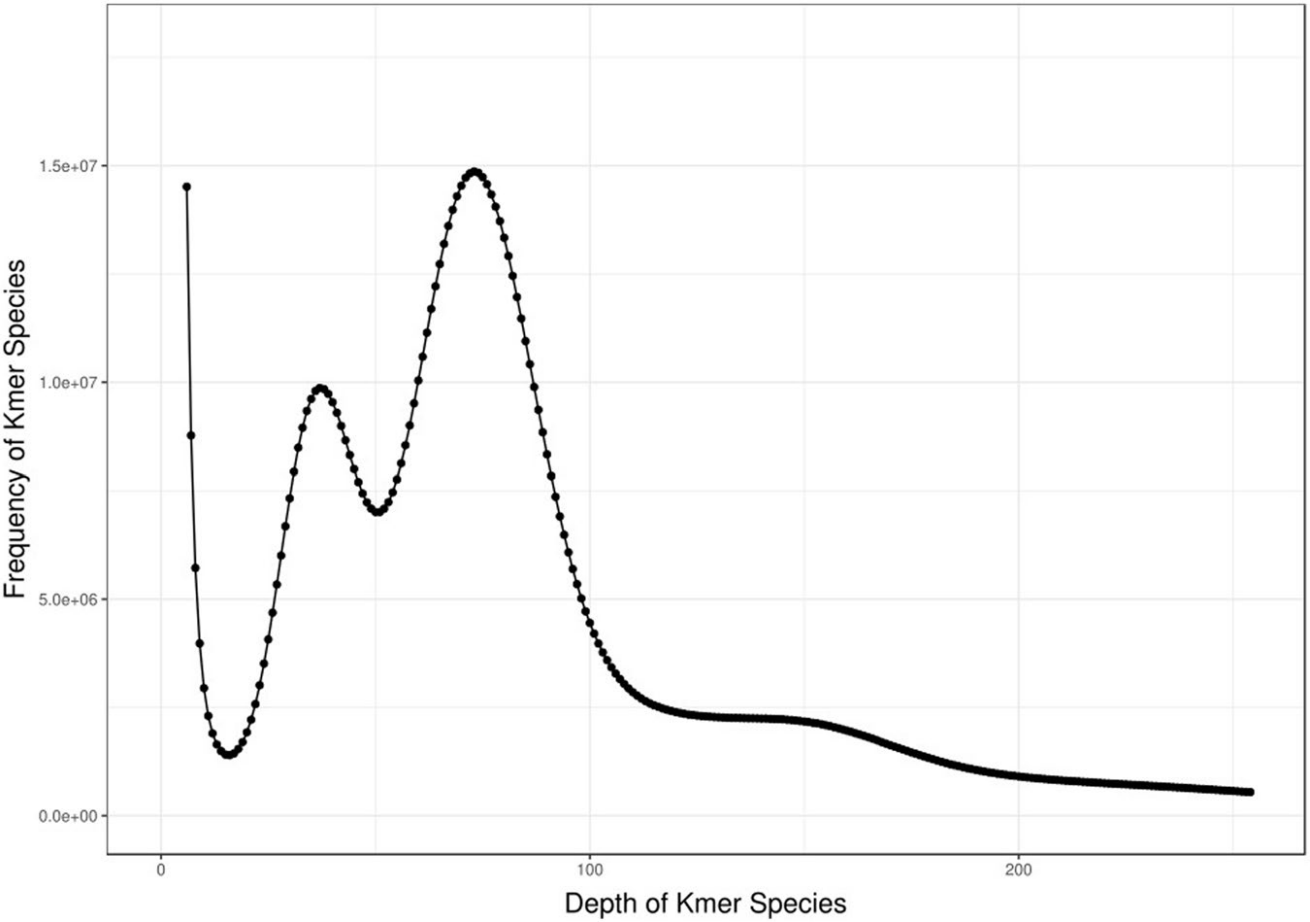
Frequency distribution of sample’s K-mer depth and K-mer species.

A K-mer-based method^30^ was applied to estimate the genome size, heterozygosity, and repeat content in *S. oleivora*. We performed a k-mer (k = 17) frequency distribution analysis using 192.1 Gb of Illumina clean data (Fig. 2). A total of 153,573,141,235 k-mers with a depth of 73 was obtained. The genome size was 2,025 Mb, the heterozygosity ratio was 0.78%, and the repeat sequence ratio was 61.37%.

### Genome assembly

PacBio Hi-Fi reads were assembled using Hifiasm(v. 0.16.1-r375) software^31^ with the default parameters. Redundant sequences were filtered out using Purge_Haplotigs (v1.0.4) software^32^ with the parameter of cutoff “-a 70 -j 80 -d 200.” Based on PacBio sequencing data, the genome length was 2090.51 Mb. The number of contigs was 302 and N50 reached 23.99 Mb. The max length was 88.20 Mb and the GC content was 34.38% (Table 2).

**Table 2:** Gene assembly results of *Sinosolenaia oleivora*.

Mode	Total length (bp)	Total number	Total number (≥2 kb)	max length (bp)	N50 (bp)	N90 (bp)	GC content (%)
hifiasm	2,127,435,208	443	443	88,197,240	22,757,865	5,864,364	34.39
hifiasm + purge_haplotigs	2,090,509,369	302	302	88,197,240	22,987,901	6,086,857	34.38

### Hi-C-assisted chromosome-level assembly

**Fig. 3:**
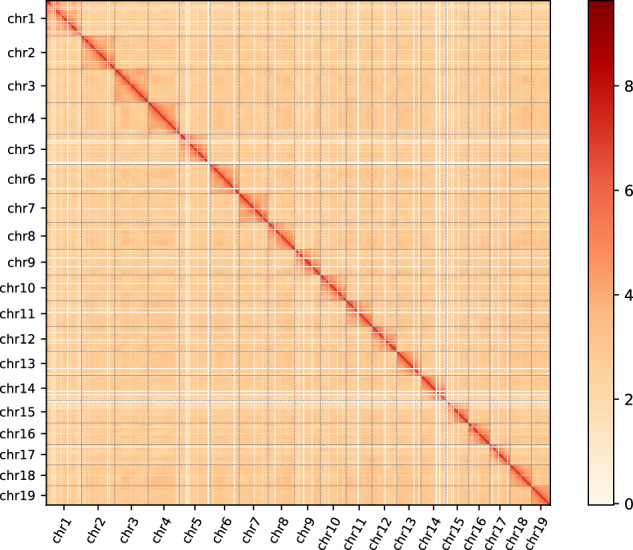
Chromosomes Hi-C heatmap of *Sinosolenaia oleivora*. Blocks represent height pseudochromosomes. The color bar represents contact density from white (low) to red (high). The same applies to Fig. 4.

**Fig. 4:**
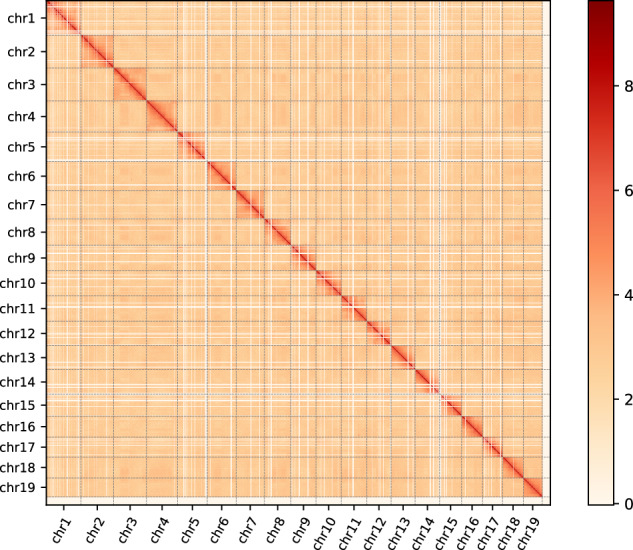
Genome-wide Hi-C heatmap of *Sinosolenaia oleivora*.

To assemble the chromosome-level genome, Hi-C sequencing data were mapped and sorted against the draft genome assembly with Juicer v1.6 software^33^. The contigs were linked to 19 distinct chromosomes by 3D-DNA (v. 180922)^34^. Based on chromosome interactions, the contig orientation was corrected and suspicious fragments were removed from the contigs in the Juicebox software^35^. The genome contigs were further anchored and oriented to chromosomes by Hi-C scaffolding. The Hi-C library generated 191.8.2 Gb of clean data, with 55.56% valid pairs. A total of 302 contigs, accounting for 98.41% of the total assembled genome, were anchored into 19 chromosomes. The 19 pseudo-chromosomes were clearly distinguished from the Hi-C heatmap with strong pseudo-chromosome interactions confirming high-quality Hi-C assembly (Figs. 3, 4). This resulted in a high-quality genome of 2052.30 Mb, with a contig N50 of 20.36 Mb and scaffold N50 of 103.57 Mb (Table 3).

**Table 3:** Statistics of Hi-C assembly results of *Sinosolenaia oleivora*.

	Sequence length (bp)	Sequence number	Contig N50 (bp)	Scaffold N50 (bp)
Draft genome	2,090,509,369	302	22,987,901	22,987,901
genome after assembly	2,052,292,908	174	20,363,756	103,572,284
Chromosome after assembly	2,019,629,721	19	20,844,760	103,572,284
Free sequence after assembly	32,663,187	155	1,348,044	1,348,044

### Repeat annotation, gene prediction, and gene functional annotation

Combined homologous and *de novo* prediction methods, repeat elements of the *S. oleivora* genome, were annotated. For homologous alignment, we used RepeatMasker (v4.1.2-p1)^36^ and Repeat-proteinmask (v4.1.0)^37^ to annotate the transposable elements (TEs) by comparing sequences to the Repbase database^38^. For *de novo* prediction, Tandem Repeat Finder (TRF) (version 4.09)^39^ was executed to detect the tandem repeat elements based on sequence features. LTR_FINDER (v. 1.07)^40^ and RepeatModeler (v. 2.0.3)^36^ were used to construct a repeat library. The library was then used to detect repetitive sequences by RepeatMasker (v. 4.1.2-p1)^36^. After eliminating redundancy, we obtained the final annotated repeat sets. A total of 1171.79 Mb repeat sequences were annotated accounting for 56.05% of the total genome sequence (Table 4). The major repetitive elements were DNA (15.74%), long interspersed nuclear elements (LINEs, 8.95%), and long terminal repeats (LTRs, 4.98%) (Table 5).

**Table 4:** Statistics of repetitive sequences in the *Sinosolenaia oleivora* genome.

Type	Repeat Size (Bp)	% of genome
Trf	342776777	16.40
Repeatmasker	306757033	14.67
Proteinmask	93857472	4.49
De novo	767880209	36.73
Total	1171787260	56.05

**Table 5:** Statistics of transposable elements for the *Sinosolenaia oleivora* genome.

Type	Repbase TEs		TE proteins		De novo		Combined TEs	
	Length (Bp)	% in genome	Length (Bp)	% in genome	Length (Bp)	% in genome	Length (Bp)	% in genome
DNA	129176254	6.18	22955138	1.1	210582215	10.07	328945510	15.74
LINE	121046511	5.79	58493281	2.8	95488865	4.57	187023002	8.95
SINE	42762259	2.05	0	0	53284731	2.55	60757406	2.91
LTR	52128004	2.49	12439501	0.6	59136256	2.83	104027479	4.98
Satellite	17082565	0.82	0	0	4940868	0.24	22003149	1.05
Simple_repeat	0	0	0	0	123320	0.01	123320	0.01
Other	106509	0.01	0	0	0	0	106509	0.01
Unknown	1938596	0.09	0	0	358766530	17.16	360311461	17.24
Total	306757033	14.67	93857472	4.49	767880209	36.73	982159858	46.98

The genome sequence was soft-masked based on repetitive element predictions and then used for protein-coding gene prediction. We employed three methods for gene prediction. For homology-based annotation, the protein sequences of *Mizuhopecten yessoensis*, *Crassostrea gigas*, *Crassostrea virginica*, and *Mytilus galloprovincialis* were downloaded from NCBI and aligned to the genome sequence using BLAST(E-value: 1e-5)^41^. Homologous sequences were then aligned to corresponding matching proteins using GeneWise (v. wise2-4-1)^42^. For the RNA-seq-based annotation, transcriptomic data were assembled using Trinity v2.11^43^, and BLAST(E-value: 1e-5)^41^ to align transcriptome to the genome. For *de novo* prediction, Augustus(v3.4.0)^44^, and Genscan (version1.0)^45^ were used to generate *de novo*-predicted gene sets. Maker (v2.31.10)^46^ was used to integrate the results from these methods to produce the final gene set. The genome sequence was also aligned to the homologous single-copy gene database of Benchmarking Universal Single-Copy Orthologs(BUSCO)^47^. MAKER (version 2.31.10)^48^ and HiCESAP (Wuhan Gooalgene Co., Ltd., https://www.gooalgene.com/) were employed to merge all the data and filter out redundancies. The combination of *de novo* and homolog-based methods predicted 22,971 protein-coding genes (Table 6). The predicted genes were functionally annotated based on exogenous protein databases including SwissProt, InterPro, TrEMBL, Kyoto Encyclopedia of Genes and Genomes (KEGG), and Gene Ontology (GO). A total of 19,229 genes, accounting for 87.52% of all predicted genes, were annotated using public databases (Table 7).

**Table 6:** Statistics of gene predictions in the *Sinosolenaia oleivora* genome.

Gene set	Number	Average gene length (bp)	Average CDS length (bp)	Average exon per gene	Average exon length (bp)	Average intron length (bp)
denovo/Genscan	51283	22222.53	1207.46	4.66	258.86	5734.67
denovo/AUGUSTUS	30749	9638.8	935.93	3.73	250.97	3188.69
homo/Mytilus_galloprovincialis	34448	15900.74	967.44	4.1	236.13	4821.79
homo/Mizuhopecten_yessoensis	19645	25436.89	1186.5	5.79	204.99	5064.79
homo/Crassostrea_virginica	20862	22987.67	1148.92	5.53	207.72	4819.84
homo/Crassostrea_gigas	20984	23083.77	1167.03	5.58	209.01	4781.42
trans.orf/RNAseq	7722	38428.06	1523.69	8.9	402.39	4411.12
BUSCO	4982	37970.19	1995.86	13.23	150.88	2942.01
MAKER	20330	32884.16	1474.92	7.37	282.29	4832.11
HiCESAP	21971	29003.27	1497.02	7.56	291.70	4081.83

**Table 7:** Functional annotations of predicted genes.

Annotated number of predicted genes	Number	Percent (%)
InterPro	15726	71.58
GO	10984	49.99
KEGG_ALL	17567	79.96
KEGG_KO	10267	46.73
Swissprot	12675	57.69
TrEMBL	18301	83.3
TF	1410	6.42
Pfam	14866	67.66
NR	18590	84.61
KOG	12091	55.03
Unannotated	2742	12.48
Annotated	19229	87.52
Total	21971	

Based on Rfam^49^ and miRbase^50^ databases, we used tRNAscan-SE (v1.3.1)^51^ to identify transfer RNAs (tRNAs), and Infernal(v1.1.2)^52^ to annotate other ncRNAs, including microRNAs (miRNAs) and small nuclear RNAs (snRNAs), and BLAST(E-value: 1e-5)^41^ was used to obtain ribosomal RNA (rRNA) to predict noncoding RNA (ncRNA) in the genome of *S. oleivora*. For non-coding RNA predictions, we successfully annotated 119 miRNAs, 2643 tRNAs, 366 rRNAs, and 867 snRNAs, with average lengths of 98, 74, 254, and 168 bp, respectively (Table 8).

**Table 8:** Non-coding RNA annotation of the *Sinosolenaia oleivora* genome.

Type		Copy	Average length (bp)	Total length (bp)	% of genome
miRNA		119	98	11611	0.000555
tRNA		2643	74	196766	0.009412
rRNA	rRNA	366	254	92902	0.004444
	18S	32	1603	51291	0.002454
	28S	24	154	3688	0.000176
	5.8S	28	154	4304	0.000206
	5S	282	119	33619	0.001608
snRNA	snRNA	867	168	145337	0.006952
	CD-box	188	173	32447	0.001552
	HACA-box	19	198	3753	0.00018
	splicing	659	165	109012	0.005215
	scaRNA	1	125	125	0.000006

### Comparative genomic analyses

**Fig. 5:**
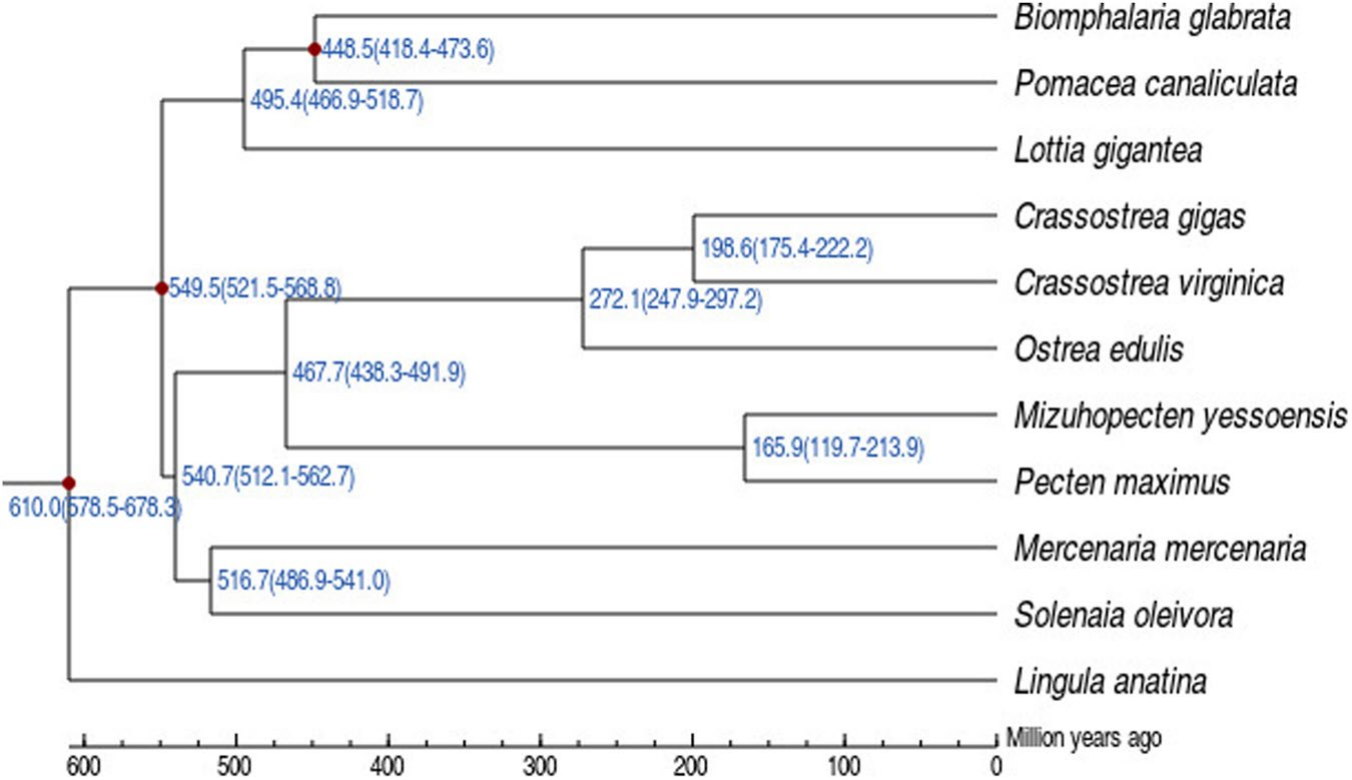
Estimates of species divergence times.

To clarify the evolutionary position of *S. oleivora*, OrthoMCL (Verison v2.0.9)^53^ with the parameter “-l 1.5” was used to detect orthologous groups by retrieving the protein sequences of *Mizuhopecten yessoensis*, *Biomphalaria glabrata*, *Crassostrea gigas*, *C. virginica*, *Lingula anatina*, *Lottia gigantea*, *Mercenaria mercenaria*, *Ostrea edulis*, *Pecten maximus*, and *Pomacea canaliculate*. Sequence alignment was performed by MUSCLE(v5)^54^ for single-copy orthologous genes. Basing on this result, KaKs Calculator(v2.0)^55^ was utilized to fetch Kolmogorov-Smirnov(Ks) with default parameters. The *S. oleivora* genome shared 82,067 gene families and 17,699 single-copy genes with ten other mollusk species. The *S. oleivora* genome contained 21971 genes clustered into 18,312 gene families and 2,273 unique families (Table 9). The phylogenetic tree was constructed using the “-f a -N 100 -m GTRGAMMA” parameter of RAxML (version 8.2.12)^56^ based on multiple sequence alignment. Divergence times were estimated using the MCMCtree (v4.9) program in PAML (v4.9)^57^ with clock = 3 and model = 0 parameters. The divergence time of *L. anatina* and *C. gigas* 619.3 (582.0–689.2 MYA); *B. glabrata* and *C. gigas* 544.1 (520.2–567.9 MYA); *P. canaliculata* and *B. glabrata* 444.6 (377.0–490.4 MYA) from TimeTree database^58^ (http://www.timetree.org/) were used for calibration. Divergence time analysis showed that *S. oleivora* was closely related to *M. mercenaria*, with a divergence time of 516.7 (486.9–541.0) Mya (Fig. 5).

**Table 9:** Gene family clustering.

Species	Gene number	Unclustered genes	Genes in families	Family number	Unique families	Unique family genes	Common families	Common family genes	Single copy genes	Average genes per family
S. oleivora	21971	3659	18312	12022	558	2273	5565	6855	1609	1.523
M. yessoensis	24450	2767	21683	16455	273	708	5565	7042	1609	1.318
B. glabrata	25308	6834	18474	11775	1007	3710	5565	7011	1609	1.569
C. gigas	31290	2475	28815	17479	640	2034	5565	7329	1609	1.649
C. virginica	34264	2313	31951	16732	728	2511	5565	8525	1609	1.91
L. anatina	26882	3188	23694	11707	1541	6041	5565	9141	1609	2.024
L. gigantea	23818	4751	19067	12389	689	3358	5565	6802	1609	1.539
M. mercenaria	36850	5163	31687	14063	1740	8049	5565	7628	1609	2.253
O. edulis	28315	2051	26264	16466	645	2173	5565	7411	1609	1.595
P. maximus	26019	2810	23209	16722	356	1032	5565	7197	1609	1.388
P. canaliculata	20881	2798	18083	11532	522	2800	5565	7126	1609	1.568

**Fig. 6:**
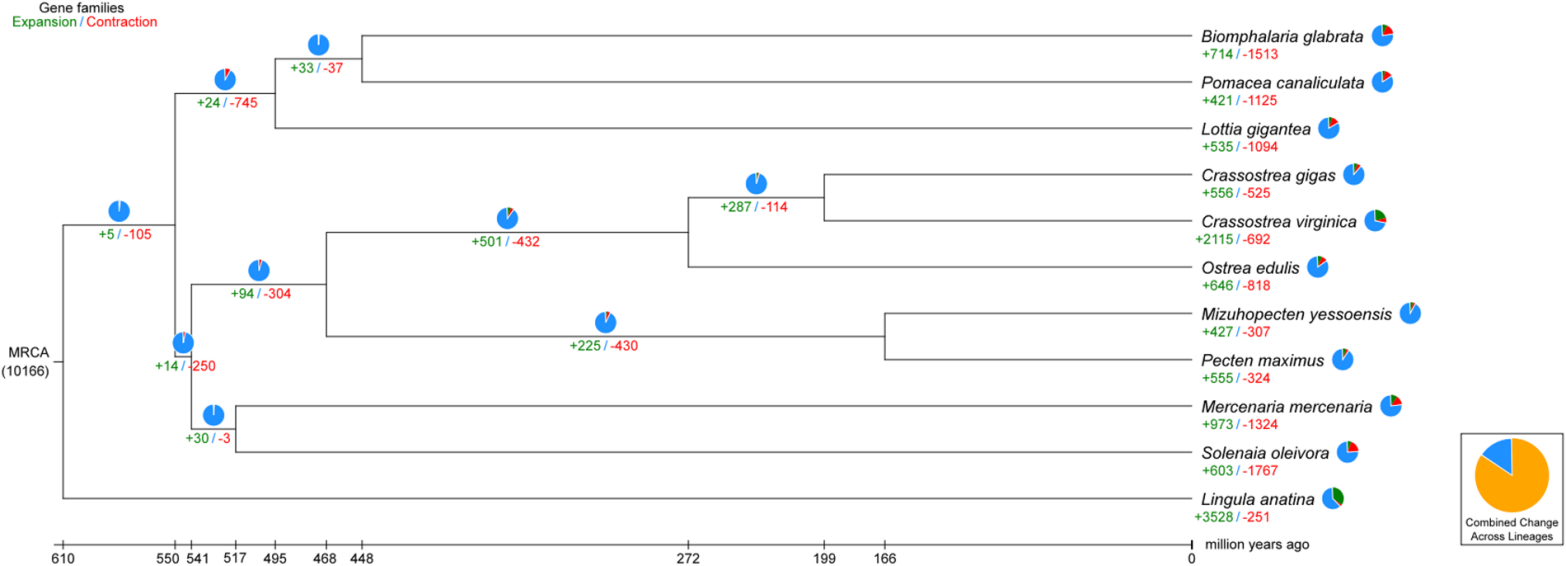
Numbers of gene families for expansion and contraction in *Sinosolenaia oleivora*. The green number represents the number of gene families that have expanded during the evolutionary process of a species, whereas the red number represents the number of gene families that have contracted.

**Fig. 7:**
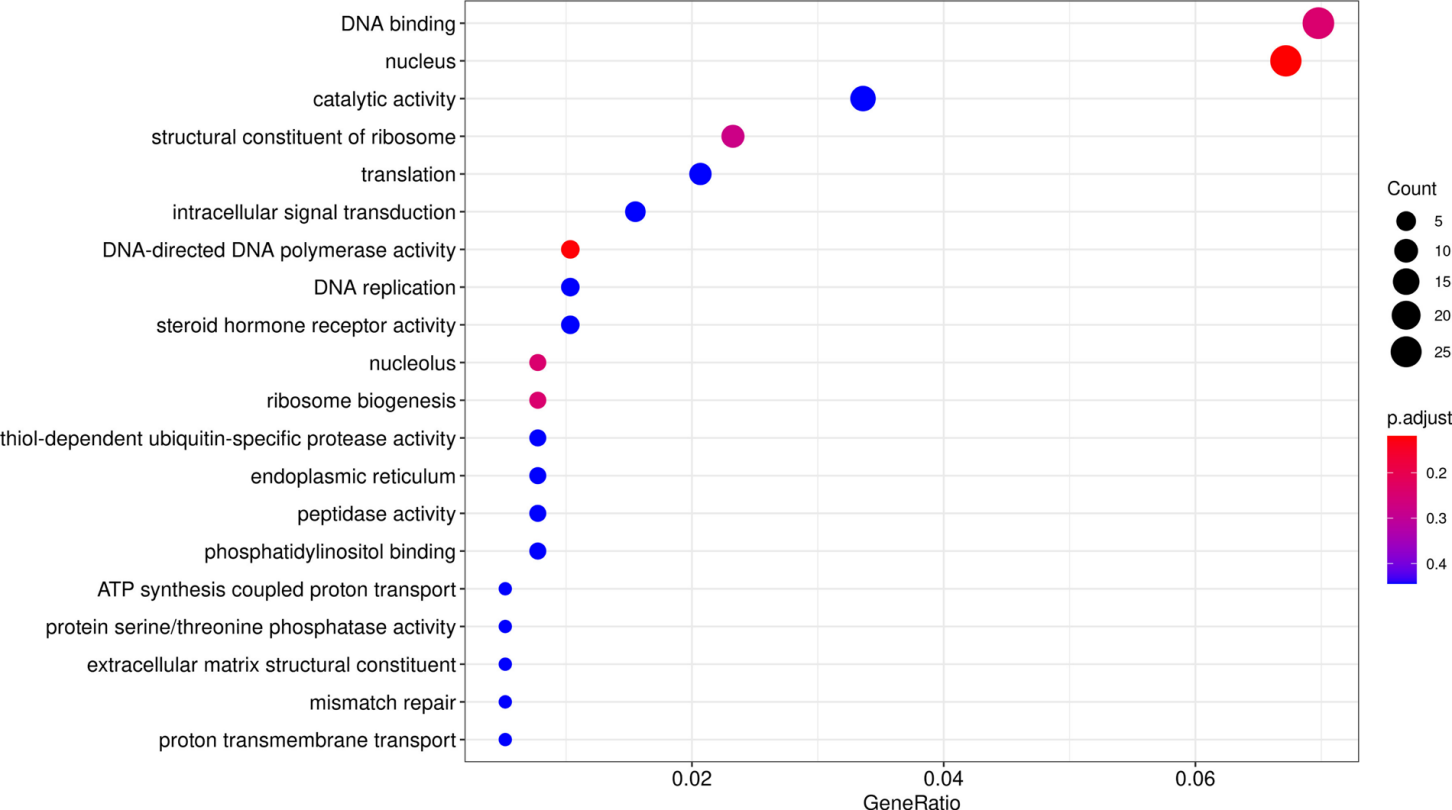
GO enrichment analysis of positively selected genes.

**Fig. 8:**
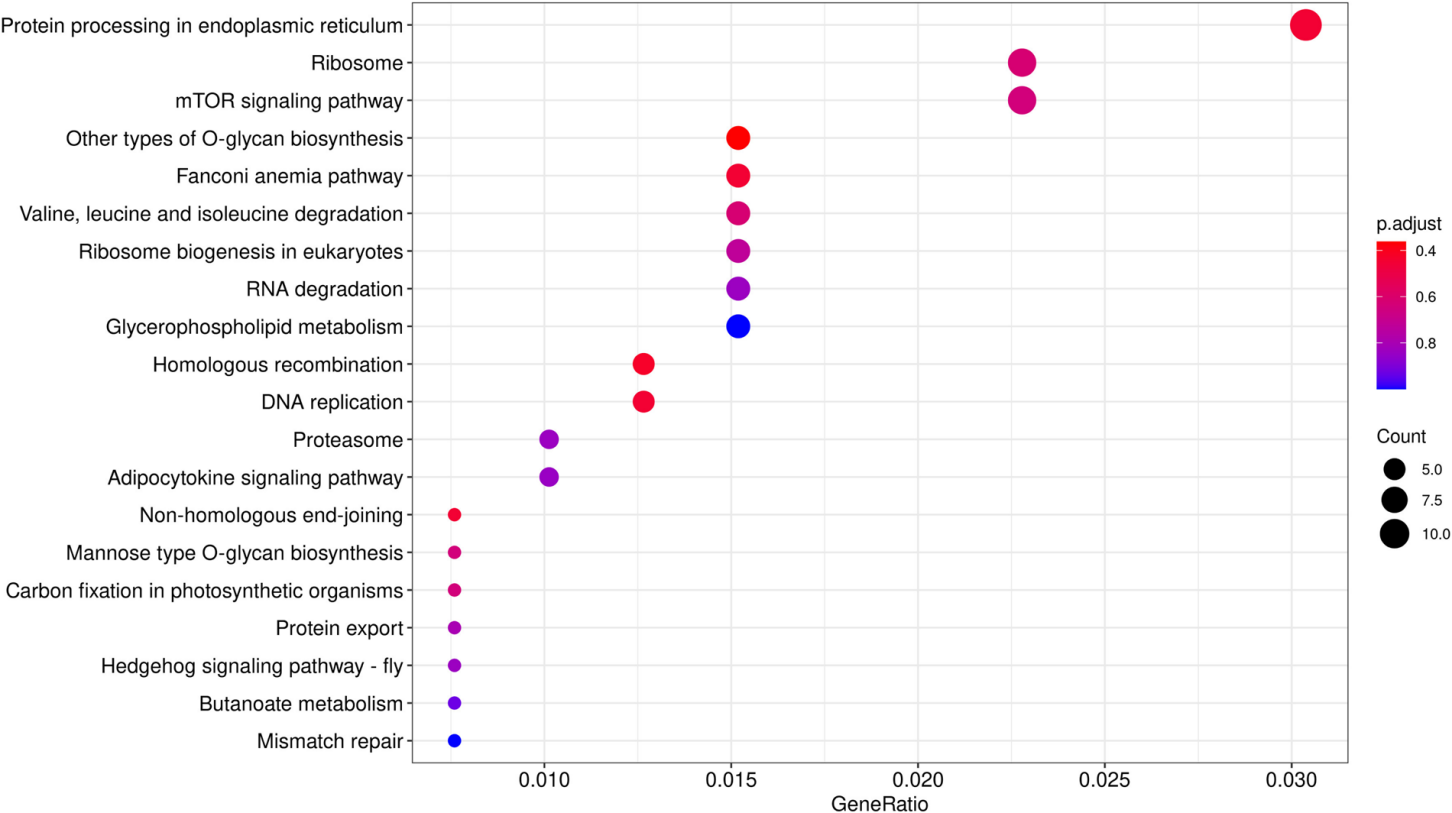
KEGG enrichment analysis of positively selected genes.

CAFE^59,60^ was applied for gene expansion and contraction analysis. Compared with the nearest ancestor, a total of 603 expanded and 1767 contracted gene families were found in *S. oleivora* (Fig. 6). There were 69 significantly expanded (984 genes) and 83 significantly contracted (118 genes) gene families (*p* < 0.05). We then performed GO and KEGG enrichment analysis and terms with enrichment-adjusted p-values ≤ 0.05 were chosen for further analysis. The program CODEML (v4.9)^57^ of PAML was used for positive selection gene (PSG) identification. PSGs were also chosen for enrichment analysis. A total of 552 protein-coding genes were positively selected in *S. oleivora* (FDR < 0.05, Table 10). GO and KEGG enrichment of positively selected genes focused on the DNA binding, nucleolus, and protein processing in the endoplasmic reticulum, ribosome, and mTOR signaling pathway (Figs. 7, 8).

**Table 10:** Protein-coding genes under positive selection in *Sinosolenaia oleivora* (FDR < 0.05).

Gene	Pvalue	FDR	Site Num
Sol0096940.1	0.041122189	1.23E-01	23
Sol0192820.1	8.40E-05	9.98E-04	12
Sol0192950.1	2.05E-05	3.06E-04	39
Sol0171310.1	0.003483956	1.93E-02	26
Sol0155040.1	0.001677492	1.12E-02	8
Sol0071120.1	7.13E-06	1.35E-04	14
Sol0023080.1	0	0.00E + 00	42
Sol0175860.1	0.015937437	6.06E-02	49
Sol0081310.1	7.31E-05	8.91E-04	7
Sol0218210.1	0.007390498	3.41E-02	12
Sol0169840.1	0.007200725	3.33E-02	5
Sol0218960.1	0.006950172	3.26E-02	4
Sol0061040.1	0.005725426	2.82E-02	11
Sol0061920.1	0.002987399	1.76E-02	7
Sol0061930.1	0.041380536	1.23E-01	4
Sol0187240.1	0.034482209	1.08E-01	6
Sol0150830.1	0.014082052	5.55E-02	2
Sol0135060.1	0.000153174	1.63E-03	7
Sol0116270.1	0.013717623	5.44E-02	7
Sol0077040.1	1.05E-05	1.88E-04	8
Sol0077020.1	0.02429022	8.21E-02	5

## Data Records

All sequencing data from three sequencing platforms have been uploaded to the NCBI SRA database (transcriptomic sequencing data: SRR28352171^61^, genomic Illumina sequencing data: SRR26551344^62^, genomic PacBio sequencing data: SRR28406055^63^, Hi-C sequencing data: SRR28406264^64^). The final chromosome-level assembled genome file has been uploaded to the GenBank database under the accession JBDPLI000000000^65^. Genome annotation files have been uploaded to the Figshare database^66^.

## Technical Validation

### Evaluating the quality of the DNA and RNA

The quality and concentration of extracted DNA/RNA were assessed using NanoDrop 2000 Spectrophotometer (Thermo Fisher Scientific, San Jose, CA, USA) and Qubit 3.0 Fluorometer (Thermo Fisher Scientific, San Jose, CA, USA)(OD260/280 and OD260/230) before the genome sequencing and their integrity was further evaluated on 1% agarose gel stained with ethidium bromide.

### Evaluating the quality of the genome assembly

We evaluated the genome assembly quality through the following measures: (i) Confirmation that the assembly result belongs to the target species was made by software BLAST(E-value: 1e-5)^26^ comparison to the NCBI nucleotide database (NT library)(Table S2, S3, Supplementary File);(ii) Illumina short reads and PacBio reads were mapped onto the assembled genome using BWA (v. 0.7.17-r1188)^67^ and Minimap2^68^ to evaluate the completeness and accuracy of the genome. The read-mapping rates were 99.27% and 99.74%, and genome coverage rates were 99.7% and 99.98% for the Illumina and PacBio reads, respectively (Table 11), indicating high mapping efficiency and comprehensive coverage. (iii) BUSCO (v5.2.3)^32^ analysis was conducted to evaluate the assembly quality based on the mollusca_odb10 database. Using BUSCO analysis, 100% (5295/5295) of complete BUSCO genes were found in the assembly, including 88.6% complete BUSCOs, 85.8% complete and single-copy BUSCOs, and 2.8% complete and duplicated BUSCOs (Table 12).

**Table 11:** The alignment of Illumina and PacBio reads to *Sinosolenaia oleivora*.

Type	Mapping rate (%)	Average sequencing depth	Coverage (%)	Coverage at least 4 × (%)	Coverage at least 10 × (%)	Coverage at least 20 × (%)
Illumina reads	99.27	97	99.7	99.45	99.2	98.75
PacBio reads	99.74	29.3	99.98	99.92	98.55	82.78

**Table 12:** BUSCO analysis results of the *Sinosolenaia oleivora* genome.

Type	Assembly		Annotation	
	Proteins	Percentage (%)	Proteins	Percentage (%)
Complete BUSCOs	4689	88.6	4575	86.4
Complete Single-Copy BUSCOs	4541	85.8	4385	82.8
Complete Duplicated BUSCOs	148	2.8	190	3.6
Fragmented BUSCOs	45	0.8	119	2.2
Missing BUSCOs	561	10.6	601	11.4
Total BUSCO groups searched	5295	100	5295	100

### Evaluating the quality of the genome annotation

BUSCO (v5.2.2)^32^ was used to evaluate the completeness of the genome annotation. The reference BUSCO database was mollusca_odb10. Among the 5295 BUSCO groups searched, 4575 (86.4%) of the complete BUSCOs were detected in the genome annotations (Table 12).

### Supplementary information

Supplementary File

## Data Availability

The manuscript did not use custom code to generate or process the data described.
