# Supplementary material for: Chromosome-level genome assembly of the freshwater mussel Sinosolenaia oleivora (Heude, 1877)
Source: Sci Data. 2024 Jun 8;11:606. doi: 10.1038/s41597-024-03451-5 (PMC11162450; doi:10.1038/s41597-024-03451-5)
Supplement: Supplementary file 1 — Supplementary File [file 41597_2024_3451_MOESM1_ESM.doc]

**Table S1 Published freshwater mussels genome assemblies**

|  | *Venustaconcha ellipsiformis* | *Margaritifera margaritifera* | *Margaritifera margaritifera* | *Unio delphinus* | *Megalonaias nervosa* | *Potamilus*  *streckersoni* | *Hyriopsis cumingii* | *Unio pictorum* |
| --- | --- | --- | --- | --- | --- | --- | --- | --- |
| Sequenced genome size (Gb) | 1.80 | 2.47 | 2.45 | 2.5 | 2.36 | 1.81 | 3.38 | 2.15 |
| Contig N50 (Mb) | 0.003 | 0.01 | 3.43 | 10.00 | 0.05 | 2.05 | 0 | 10.61 |
| Scafold N50 (Mb) | 0.006 | 0.29 | 3.43 | 10.91 | 0.05 | 2.05 | 3.19 | 10.61 |
| GC content (%) | 34.19 | 35.42 | 35.30 | 35.07 | 35.82 | 33.79 | 36.07 | 34.82 |
| Complete BUSCOs (%) | 68.00 | 86.80 | 99.2 | 98.5 | 83.00 | 94.6 | 93.00 | 99.20 |
| Fragmented BUSCOs (%) | 21.00 | 5.90 | 0.40 | 1.60 | 9.00 | 1.20 | 3.10 | 0.80 |
| Duplicated BUSCOs (%) | 1.00 | 1.00 | 1.60 | 2.40 | 2.10 | 0.90 | 0.80 | 3.10 |

**Table S2 Comparison of reads with NT libraries**

| Species | Blast number | Total blast number | Total (%) |
| --- | --- | --- | --- |
| *Hyriopsis* | 174 | 500 | 34.8 |
| *Solenaia* | 90 | 500 | 18 |
| *Mastacembelus* | 28 | 500 | 5.6 |
| *Rhinatrema* | 27 | 500 | 5.4 |
| *Cristaria* | 19 | 500 | 3.8 |
| *Elliptio* | 15 | 500 | 3 |
| *Pomacea* | 9 | 500 | 1.8 |
| *Thalassophryne* | 8 | 500 | 1.6 |
| *Oryzias* | 6 | 500 | 1.2 |
| *Tachysurus* | 5 | 500 | 1 |
| *Unio* | 5 | 500 | 1 |
| *Pleurobema* | 5 | 500 | 1 |
| *Betta* | 4 | 500 | 0.8 |
| *Darwinula* | 4 | 500 | 0.8 |
| *Caenorhabditis* | 4 | 500 | 0.8 |

**Table S3 Comparison of interrupted sequences with NT libraries**

| Genus | Blast number | Total blast number | Total (%) |
| --- | --- | --- | --- |
| *Hyriopsis* | 48,042 | 119,102 | 40.34 |
| *Elliptio* | 19,928 | 119,102 | 16.73 |
| *Solenaia* | 10,492 | 119,102 | 8.81 |
| *Cristaria* | 9,072 | 119,102 | 7.62 |
| *Rhinatrema* | 3,973 | 119,102 | 3.34 |
